# Topotecan synergizes with CHEK1 (CHK1) inhibitor to induce apoptosis in ovarian cancer cells

**DOI:** 10.1186/s12885-015-1231-z

**Published:** 2015-03-28

**Authors:** Marianne K Kim, Jana James, Christina M Annunziata

**Affiliations:** Women’s Malignancies Branch, Center for Cancer Research, National Cancer Institute, NIH, Bethesda, MD 20892 USA

**Keywords:** Topotecan, CHEK1 (CHK1), Ovarian cancer

## Abstract

**Background:**

Topotecan (TPT) is a therapeutic option for women with platinum-resistant or -refractory ovarian cancer. However, the dose-limiting toxicity of TPT is myelosuppression. This led us to seek a combination treatment to augment TPT anti-cancer activity in a cancer-targeted manner. Ovarian serous cancers, a major subtype, show dysregulated DNA repair pathway and often display a high level of CHEK1 (CHK1), a cell cycle regulator and DNA damage sensor. CHEK1 inhibitors are a novel approach to treatment, and have been used as single agents or in combination chemotherapy in many cancers.

**Methods:**

We evaluated the cellular effects of TPT in a panel of high grade serous (HGS) and non-HGS ovarian cancer cells. We then determined IC50s of TPT in the absence and presence of CHEK1 inhibitor, PF477736. Synergism between TPT and PF477736 was calculated based on cellular viability assays. Cytotoxic effect of the combined treatment was compared with apoptotic activities by Caspase3/7 activity assay and Western blotting of cleaved-PARP1 and γH2AX.

**Results:**

Non-HGS ovarian cancer cells were generally more sensitive to TPT treatment compared to HGS ovarian cancer cells. When combined with CHEK1 inhibitor, TPT potently and synergistically inhibited the proliferation of HGS ovarian cancer cells. This dramatic synergism in cellular toxicity was consistent with increases in markers of apoptosis.

**Conclusions:**

Our findings suggest that the addition of CHEK1 inhibitor increases the response of ovarian cancer cells to TPT. Furthermore, reduced dosages of both drugs achieved maximal cytotoxic effects by combining TPT with CHEK1 inhibitor. This strategy would potentially minimize side effects of the drugs for extended clinical benefit.

**Electronic supplementary material:**

The online version of this article (doi:10.1186/s12885-015-1231-z) contains supplementary material, which is available to authorized users.

## Background

Ovarian cancer consists of 4 major histologic subtypes including serous, clear cell, endometrioid, and mucinous [1]. Each harbors defining patterns of molecular aberrations that could affect response to treatment and clinical outcome. Most ovarian cancers are diagnosed at advanced III or IV stage, and current standard of care includes surgical cytoreduction and platinum-based chemotherapy. Ovarian cancer has a high response rate, but most cancers eventually relapse and metastasize, becoming resistant and refractory to standard chemotherapy.

The most common subtype, high-grade serous (HGS) ovarian cancer, is characterized by defective DNA repair [2]. Treatment approaches commonly include chemotherapies that induce DNA damage, since the ovarian cancer cell will die when it is incapable of recovering from injury to its DNA. Platinum chemotherapies, the hallmark of DNA damaging agents, have been combined with other cancer drugs such as taxanes, which alter microtubule function [3]. Topoisomerase I and II inhibitors, such as camptothecin and etoposide, block DNA unwinding during replication, transcription, and chromatin remodeling, by preventing the rejoining process of the DNA strands and resulting in accumulation of DNA breaks. In particular, the topoisomerase I inhibitor topotecan (TPT) is active against many cancer types including platinum-sensitive and resistant ovarian cancers [4,5]. Unfortunately, side effects of TPT and other DNA-damaging agents include low blood counts, nausea, vomiting, diarrhea, and fatigue at doses that are required to induce tumor responses. These adverse events limit the dose that can be administered and the duration of treatment. Ideally, combination therapy with TPT should include agents that exploit defects in the cancer cells to augment tumor-specific cell death without increasing intolerable adverse effects to the patient.

Throughout the lifetime of a cell, DNA damage is introduced either exogenously or endogenously. Cellular sensing of DNA damage and its repair occur throughout cell cycle checkpoints. For example, p53 is a main checkpoint regulator in G1/S phase, while checkpoint kinase 1/2 (CHEK1/2, CHK1/2) is crucial in S and G2/M checkpoints. In HSG ovarian cancers, p53 is either null or mutated, increasing the cellular dependency on CHEK1/2 for DNA damage repair and survival. Supporting this notion, we recently showed that *CHEK1* gene is overexpressed in nearly all cases of HGS ovarian cancer as compared to normal ovarian surface epithelium in The Cancer Genome Atlas (TCGA) dataset, suggesting that CHEK1 is required for cells to tolerate the defective DNA repair intrinsic to HGS cancers [2,6]. Upon DNA damage, CHEK1 is activated by ATR signaling and sequentially phosphorylated at residues S317 and S345 which subsequently induce autophosphorylation on S296 to trigger cell cycle arrest and DNA repair [7]. Indeed, recent studies suggested that both pS345 CHEK1 and pS296 CHEK1 could be used as pharmacodynamic markers to monitor the CHEK1 inhibition upon treatment with CHEK1 inhibitor alone and combination treatments [8,9].

Inhibition of CHEK1/2 could therefore present a novel therapeutic strategy. Cancer cells with dysfunctional p53 may be particularly susceptible to such drugs. CHEK inhibition would cause premature entry into mitosis without adequate repair of DNA. This excessive DNA damage would lead to mitotic catastrophe and subsequent cancer cell death. For this reason, several CHEK1 inhibitors are under development as single agents as well as a combined therapy [10,11].

Since TPT is used as therapy for recurrent cisplatin resistant and refractory ovarian cancer, and increased expression levels of *CHEK1* are observed in ovarian cancers, we hypothesized that inhibiting CHEK1 could be a means to enhance the anti-cancer activity of TPT preferentially in ovarian cancer cells, thus increasing their therapeutic index in cancer cells as compared to normal tissues.

## Methods

### Cell lines

Ovarian cancer cell lines, A2780, HeyA8, Igrov1, Skov3, Ovcar5, Ovcar3, Ovcar8, OV90, PEO1 and PEO4 were maintained in RPMI supplemented with 10% heat-inactivated FBS and 1X Pen/Strep.

### Chemical inhibitors

Stock solutions of Topotecan HCl (Selleck, S1231) and PF477736 (Selleck, S2904) were prepared in DMSO and aliquots were stored at −80°C.

### XTT assay

Cells were seeded in 96-well plates at a density of 1–2,000 cells/50 μl/well in triplicate. In general, the drug was added 24 hours after seeding and XTT assay was routinely performed in 3 days after drug treatment unless indicated. Cellular viability was assessed by incubating cultures with XTT freshly mixed with PMS (Sigma) and absorbances were read in a Tecan plate reader (Research Triangle Park, NC). Cellular proliferation was calculated relative to experimental negative controls and standard deviation was calculated from triplicates.

### The cancer genome atlas data

TCGA ovarian cancer dataset was analyzed and extracted using a web-based tool (http://www.cbioportal.org/public-portal/) [2].

### Western blot analysis

Total protein was extracted from OC cell lines with 1% NP40 lysis buffer containing 150 mM NaCl, 50 mM TrisHCl, 10% glycerol, 1 X Halt proteinase inhibitor cocktail, 5 mM NaF, and 1 mM NaOrthovanadate. Protein concentrations were estimated using BCA Protein Assay Kit (Thermo Scientific, Rockford, IL). The proteins were separated on the NuPage 4–12% gel (Invitrogen, Carlsbad, CA) and the band was visualized using either Lumina Classico or Crescendo Western HRP substrate system (Millipore) depending on the signal intensities. Antibodies Chk1 G-4 (Santa Cruz, sc-8408), phospho-Chk1 Ser345 (Cell Signaling, #2348), phospho-Chk1 Ser296 (Cell Signaling, #2349), cleaved PARP (Cell Signaling, #9541), phospho-H2AX (Cell Signaling, #5438), Topoisomerase I (Abcam, ab3825), and GAPDH (Millipore, MAB374) were used in this study, and the secondary antibodies ECL anti-rabbit IgG HRP and ECL anti-mouse IgG HRP (GE Healthcare) were used at 1:5000 dilutions.

### Caspase3/7 assay

Cells were seeded in 96-well white-walled plates at a density of 2,000 - 5,000 cells/50 μl/well and the drug in a 50 μl volume was added 24 hours after seeding. The caspase activity was measured after 16 hours additional incubation followed by adding 40 μl Caspase-Glo reagent (Promega) per well.

### Cell cycle analysis

Cells were seeded at 8 X 10^5^/60 mm plate and 8 hours prior to drug treatment. Fresh complete medium containing 0.2 μM TPT, 0.5 μM PF477736, or both drugs was added and cells were incubated for 16 hours. The procedure was performed according to manufacturer’s protocol (BD Pharmingen APC BrdU Flow kit). Following 30 minutes incubation after adding BrudU, cells were trypsinized and collected for subsequent fixation and permeabilization. Cells were analyzed by FACS Calibur (Becton Dickinson) after staining with APC-anti-BrdU and 7-AAD. Cell cycle was analyzed using FlowJo software.

## Results

### HGS ovarian cancer cells are more resistant to TPT treatment than non-HGS cells

**Figure 1:**
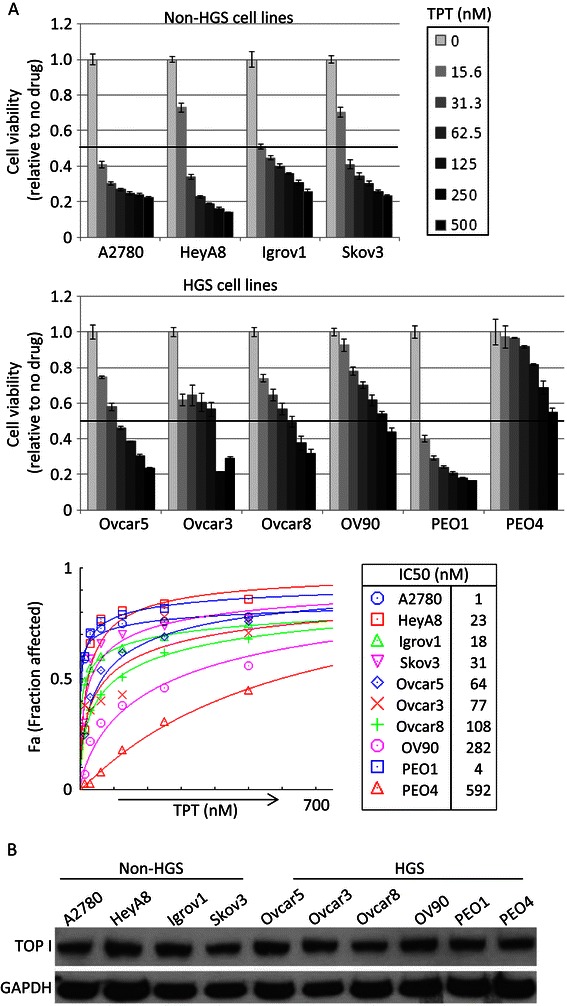
**Pharmacological inhibition of Topoisomerase I in ovarian cancer cell lines. A**. TPT toxicities were measured by XTT assay. Cells were seeded at 1000 cells/well in 50 μl and 3 replicates 16–20 hours prior to the addition of the drug. XTT assay was performed 3 days later upon drug treatment. The viability was calculated relative to no drug treatment and the error bars represent standard deviations calculated from 3 replicates. Shown is a representative of 2 – 5 independent experiments depending on cell lines. IC50 values were calculated by CompuSyn after converting relative viability values to fraction affected numbers. **B**. The expression level of Topoisomerase I was examined using total lysates by Western blotting. GAPDH was used as a loading control.

A recent study proposed HGS ovarian cancer model cell lines based on the comparison of genomic profiles of ovarian cancer cell lines with HGS ovarian tumors [12]. We initially determined cytotoxicity of TPT in a panel of 4 non-HGS, and 6 HGS ovarian cancer cell lines including PEO1 and PEO4, a pair of HGS ovarian cancer cell lines established from the same patient before and after platinum-based chemotherapy [13]. In general, all HSG ovarian cancer cells except PEO1 were more resistant to TPT treatment compared to non-HGS ovarian cancer cell lines (Figure 1A). When IC50 values were determined by CompuSyn software, PEO1 cells showed IC50 of less than 10 nM, comparable to those of non-HGS ovarian cancer cells. Interestingly, OV90 and PEO4 were the most resistant, showing approximately 50% of cellular viability even at 500 nM. Next, we investigated the relationship between the expression levels of Topoisomerase I and the sensitivities to TPT among these ovarian cancer cell lines. At steady-state levels, both non-HGS and HGS cell lines showed similar levels of topoisomerase I (Figure 1B). This suggests that the steady state level of topoisomerase cannot predict the sensitivity to TPT. Nonetheless, HGS ovarian cancer cells were generally less sensitive to TPT treatment than non-HGS ovarian cancer cells.

### *TOP1* is altered in a subset of ovarian serous tumors with an association with a poor overall survival

**Figure 2:**
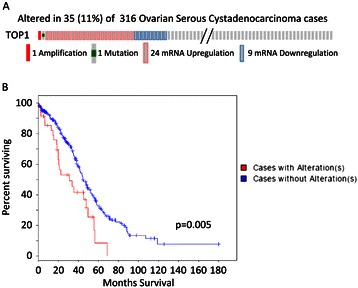
**Analysis of*TOP1*in TCGA dataset. A**. *TOP1* alterations including copy number, mutation, and mRNA expression were examined. **B**. Overall survival analysis based on TOP1 alterations was extracted from TCGA data analysis.

In TCGA, ovarian cancer patient samples showed alterations in the gene for topoisomerase I, *TOP1* [2]. Thirty five cases out of 316 ovarian serous cancer contained *TOP1* alterations including 1 amplification, 1 mutation, and 33 mRNA dysregulations (Figure 2A). These alterations were significantly associated with a worse overall survival compared to the cases without alterations (Figure 2B). Interestingly, 9 cases showed down-regulation of *TOP1* mRNA. We also searched the mutation status of *TOP1* gene in the Cancer Cell Line Encyclopedia (CCLE) [14] and found no alterations of *TOP1* in 51 ovarian cancer cell lines, but identified 29 mutations among over 1000 cancer cell lines in that dataset (Additional file 1: Table S1). While *TOP1* mutations were rare in ovarian cancer cell lines and tumors, a subset of ovarian cancers had dysregulated expression levels of *TOP1*. The correlation with poor survival suggests a mechanism of resistance to DNA damaging chemotherapy, since the patient tumors were taken at the time of initial diagnosis. These alterations could similarly serve as a biomarker for poor clinical response to TPT. Therefore, we proceeded to identify compounds that would increase the anti-cancer activity of TPT in HGS ovarian cancer cells.

### Co-treatment of TPT and PF477736 synergistically kills ovarian cancer cells

**Figure 3:**
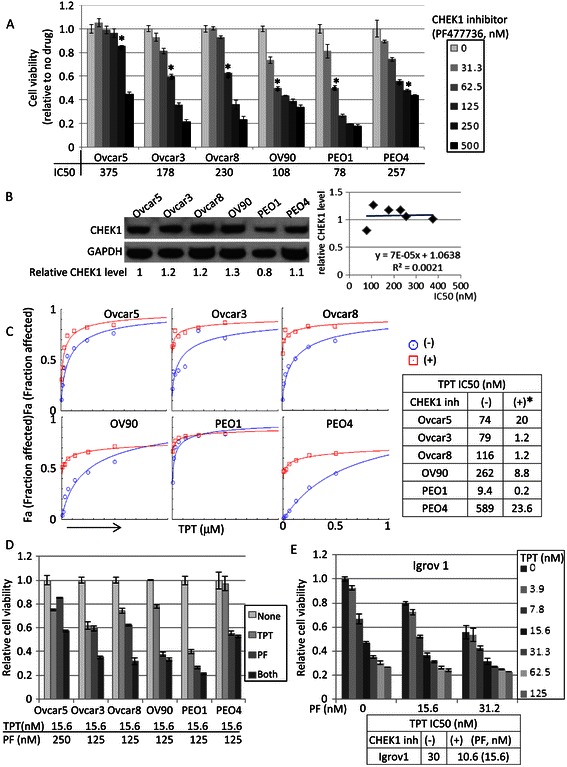
**Cytotoxicity of TPT was increased in the presence of CHEK1 inhibitor. A**. IC50 values of CHEK1 inhibitor (PF477736) were determined by XTT assay done in 3 days upon treatment in 3 replicates. (*) indicates the concentrations used in Figure 3C. **B**. Western blot analysis of total CHEK1 was done using total protein lysates. GAPDH was used as a loading control. The band intensity was quantified by ImageJ and CHEK1 level was normalized by GAPDH. The CHEK1 levels in different cell lines were calculated relative to that of Ovcar5. IC50 values and relative CHEK1 levels were plotted to examine their relationships by Microsoft Excel. **C-E**. XTT assays were done in the absence and presence of CHEK1 inhibitor in 3 replicates and Fa (fraction affected), fraction of dead cells, was calculated from XTT assay. TPT IC50 values were calculated by CompuSyn software.

Since HGS ovarian cancer cells required higher concentrations of TPT than non-HGS cells, we sought to identify a combination that achieved maximal cytotoxic effect with a minimal dose of TPT. We previously found that *CHEK1* was overexpressed in most HGS ovarian cancers [6], and hypothesized that inhibition of CHEK1 would increase the therapeutic index of TPT, given its importance in DNA damage repair pathways. Therefore, we investigated whether CHEK1 inhibitor, PF477736, sensitized HGS ovarian cancer cell lines to TPT treatment. PF477736 is a selective ATP-competitive inhibitor that can enhance the antitumor activity of gemcitabine [15]. First, the IC50 values of PF477736 were determined by XTT assays. All 6 cell lines showed decreases in cellular viabilities in a dose dependent manner with a range of IC50 78–375 nM (Figure 3A). We found no clear relationship between total CHEK1 protein level and sensitivity to CHEK1 inhibitor (Figure 3B); most HGS cell lines, however, expressed abundant levels of CHEK1. Of note, ovarian cancer cell lines used in this study express either mutated or no p53, except A2780 (*p53 wt*). Specifically, Ovcar5 and Skov3 do not express p53, while HeyA8, Igrov1, Skov3, Ovcar3, Ovcar8, Ovcar90, PEO1 and PEO4 express mutant p53. Therefore, CHEK1 inhibitor sensitivity is not correlated with the status of p53 in ovarian cancer cell lines. With sub-lethal concentrations of CHEK1 inhibitor indicated in Figure 3A (*), the dose–response to TPT was significantly shifted in 6 HGS ovarian cancer cell lines. The range of IC50 was from 9.4 nM to 589 nM in the absence of PF477736, and shifted downward more than 10-fold (between 0.2 nM to 23.6 nM) when CHEK1 activity was inhibited (Figure 3C). Synergism between CHEK1 inhibitor and TPT was calculated using a 6 X 6 matrix format with 2-fold serial dilutions of both drugs at a 1:2 ratio of TPT:PF (Table 1). Combination Index (CI) values below 1 were achieved in all cell lines, indicating synergism between these drugs (Table 1). Since PEO1 cells were highly sensitive to single treatment with either TPT or PF477736 (Figure 1A, Figure 3A), the maximal combined cytotoxicity was achieved at concentrations 2 or 4 fold less than in other cell lines (with 63 nM of TPT and 125 nM of PF477736). Although TPT combination with CHEK1 inhibitor decreased TPT IC50 values by 30- and 25-fold, respectively, in OV90 and PEO4 cells, complete cytotoxicity was not achieved (Figure 3C). When plotted at a minimal cytotoxic concentration of TPT (15.6 nM), cell survival was generally further decreased when combined with the indicated sub-lethal concentrations of CHEK1 inhibitor (Figure 3D). In order to examine whether PF477736 also potentiates TPT activity in non-HGS cells, we measured the cytotoxicity in Igrov1 cells upon co-treatment with PF477736 and TPT using lower concentrations of both inhibitors than in HGS cells (Figure 3E). As expected, the IC50 of TPT was decreased from 30 to 10.6 nM in the presence of 15.6 nM of PF477736, although the potentiation was not as dramatic as in HGS ovarian cancer cells.

**Table 1:** **Combination index (CI) values of topotecan and PF477736 co-treatment in ovarian cancer cells**

Total Dose (uM)	Ovcar5		Ovcar3		Ovcar8		OV90		PEO1		PEO4	
TPT + PF	Fa	CI	Fa	CI	Fa	CI	Fa	CI	Fa	CI	Fa	CI
0.25 + 0.5	0.84	1.4	**0.86**	**0.9**	0.87	1.6	0.71	1.2	0.85	1.9	0.64	1.3
0.125 + 0.25	**0.79**	**0.9**	0.83	0.5	**0.83**	**0.9**	**0.67**	**0.9**	0.84	1.1	**0.60**	**0.8**
0.063 + 0.125	0.70	0.6	0.73	0.5	0.74	0.5	0.60	0.7	**0.81**	**0.7**	0.54	0.5
0.032 + 0.063	0.47	0.7	0.76	0.2	0.68	0.3	0.54	0.5	0.77	0.5	0.42	0.4

Fa (Fraction affected) was calculated from XTT assays and Fa value of greater than 0.8 is cytotoxic considering the detectable limit of XTT assay. Cellular killing was examined under light microscope before measuring the viability. Synergistic CI values with Fa values are shown in bold.

**Figure 4:**
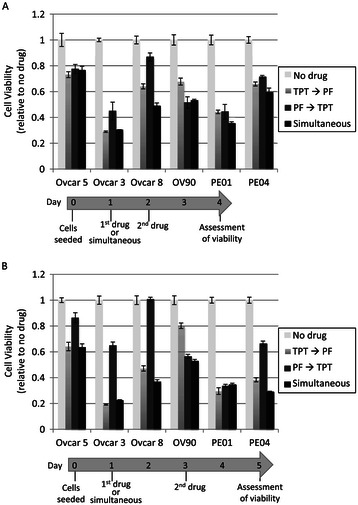
**Simultaneous treatment with TPT and PF477736 was generally more potent than sequential treatments of individual drugs.** Treatment schedules of 24 **(A)** and 48 **(B)** hours apart between drugs are shown. The concentrations of TPT and PF477736 were 10 nM and 0.1 μM, respectively, except Ovcar5 (0.3 μM PF477736). Cellular viability was measured by XTT assay in 3 replicates and calculated relative to untreated cells.

We next investigated whether the order of drug administration (sequential or simultaneous) affected the synergism achieved with TPT and PF477736. Ovarian cancer cells were treated with two drugs simultaneously or sequentially, either 24 or 48 hours apart. In general, simultaneous treatment was more effective than sequential exposure, regardless of the order of dosing, treatment duration or time of exposure (Figure 4). Overall, combined simultaneous treatment with TPT and PF477736 synergistically killed ovarian cancer cells.

### Co-treatment with TPT and CHEK1 inhibitor increases apoptosis and DNA damage response

**Figure 5:**
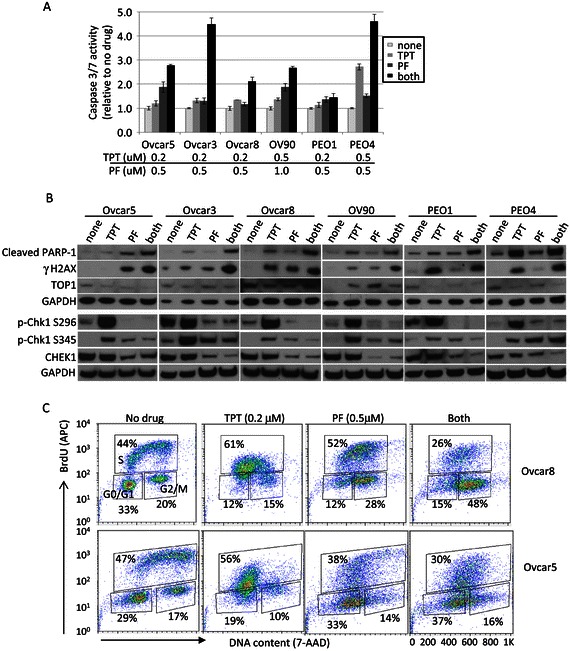
**Combined treatment with TPT and PF477736 showed increased apoptotic activities and DNA damage. A**. Apoptotic activity was measured by Caspase3/7 activity assay. Cells were treated with TPT and/or PF477736 for 16 hours with indicated drug concentrations and Caspase3/7 activity was measured relative to untreated samples in triplicates. **B**. Cells were treated at the same concentrations as in Caspase3/7 assay for 16 or 24 hours and total cell lysates were prepared for Western blotting. GAPDH was used as a loading control. **C**. Eight hundred thousand cells were plated in 60 mm plates 8 hours prior to drug treatment. Cells were treated for 16 hours with TPT (0.2 μM) and/or PF (0.5 μM). G_0_/G_1_, S, and G_2_/M phases were measured based on staining of APC-BrdU and 7-AAD by flow cytometry. Cell cycle was analyzed by FlowJo software.

Given the known mechanisms of each drug, we next measured apoptosis and DNA damage response upon single and dual exposure. Caspases 3 and 7 are activated in the final stages of apoptosis and cleave PARP. As expected, caspase3/7 activity was consistently the most elevated by combined treatment with TPT and PF477736 (Figure 5A). Cleaved PARP-1 level, a marker of late apoptosis, was increased accordingly (Figure 5B). DNA damage is indicated by phosphorylation of H2AX, and this γ-phosphorylation was increased most highly with combination treatment in all cell lines (Figure 5B). Each agent alone and co-inhibition triggered CHEK1 S345 phosphorylation. TPT treatment significantly induced CHEK1 S296 autophosphorylation, a marker for CHEK1 activity, but the autophosphorylation was clearly decreased upon PF477736 alone and combination treatments. Total CHEK1 level was decreased upon PF477736 alone and co-inhibition, and total topoisomerase I level was decreased upon TPT alone and co-inhibition (Figure 5B). In summary, DNA damage was greatest with co-treatment in all 6 cell lines, and the activation of CHEK1 activity upon TPT treatment was abrogated by co-treatment with PF477736. CHEK1 activation upon TPT treatment may potentially provide the resistance and relapse to TPT monotherapy especially in CHEK1-overexpressing HGS ovarian cancer patients.

We also performed cell cycle analysis in both p53 null (Ovcar5) and p53 mutant (Ovcar8) cell lines to investigate whether inhibition of CHEK1 with PF477736 affects TPT-induced cell cycle arrest. In both cell lines, we observed that PF477736 abrogated TPT-induced cell cycle arrest (Figure 5C).

Taken together, co-treatment with TPT and PF477736 more potently induced DNA damage and apoptosis than either single treatment alone in ovarian cancer cells.

## Discussion

We recently found that *CHEK1* gene was overexpressed in most of HGS ovarian cancer, and combined inhibition of pro-survival modulator, IKKε, cooperatively induced apoptosis in ovarian cancers [6]. In this study, we have further extended this finding to evaluate CHEK1 inhibitor-based combination therapy. TPT is currently used for the salvage treatment of ovarian cancer, but myelosuppression is the dose-limiting toxicity. High doses of TPT are poorly tolerated, resulting in treatment delays or dose reductions. Therefore, we hypothesized that combining TPT with CHEK1 inhibitor may be an effective and feasible approach to maximize the therapeutic index between cancer cells and normal, allowing lower doses of both agents in order to minimize the dose-limiting toxicity. Here, we found that HGS ovarian cancer cell lines generally required higher concentrations of TPT, in the absence of CHEK1 inhibition, to achieve equivalent cytotoxicity compared to non-HGS ovarian cancer cell lines. When combined with CHEK1 inhibitor (PF477736), TPT synergistically produced maximal cytotoxic effects at reduced dosages.

Based on its mechanism of action, it has been hypothesized that CHEK1 inhibition would potentiate the effects of standard chemotherapy in a wide range of cell lines. In particular, CHEK1 inhibitors (e.g. V158411, AZD7762, MK-8776, GNE-900, CCT244747) have been combined with camptothecin and non-camptothecin inhibitors of topoisomerases [8,16-19]. While combined inhibitions generally showed synergistic effects, a study showed limited increase in cancer cell death with MK8776, reporting that *CHEK1* depletion with siRNA or inhibition with MK-8776 only minimally sensitized Ovcar8 cells to TPT in colony formation assays [17]. Combination of GNE-900 and camptothecin was minimally effective than either alone *in vivo* xenograft model [18]. The reasons for this discrepancy may be due to different potency and penetrance of CHEK1 inhibitors as well as different measurements in biological assays.

Our current study provides insight into the nuances of how the combination of CHEK1 and topoisomerase inhibition may be most effectively used. Not all ovarian cancer cell lines were equally sensitive to either agent alone or in combination. Our data demonstrate that these agents may have the most therapeutic potential in HGS ovarian cancers, where the combination demonstrated the greatest degree of synergy. In addition, the order of drug administration was relevant, with the most cell death occurring when both drugs were given together, or when TPT was given first, followed by PF477736. Interestingly, the least effective sequence was administering PF477736 prior to TPT. Similarly, a study using CHEK1 inhibitor AZD7762 showed that gemcitabine administration either concurrent with or before AZD7762 resulted in maximal chemosensitization in pancreatic cancer model [9].

Several CHEK1 inhibitors (PF477736, MK-8776/SCH900776, and LY2606368) have completed phase 1 evaluation as single agents, but formal reports are not available. In addition, PF477736 (Pfizer) was evaluated in advanced solid tumors in combination with gemcitabine (NCT00437203) and was discontinued from development. CHEK1 inhibitor MK8776 (Merck) in combination with gemcitabine has also reported phase 1 evaluation in patients with solid tumors. Combining an anti-metabolite (gemcitabine) with MK8776 was well tolerated with few patients experiencing grade 3–4 adverse events, the most common being neutropenia (15%) [20].

TPT has been combined clinically with angiogenesis inhibitors bevacizumab or trabananib [21,22]. Such a strategy combines agents targeting two separate pathways (DNA replication and angiogenesis) important to cancer, without overlapping side effects so as not to limit dosage of either drug. The combination of TPT with CHEK1 inhibitor takes a different approach, by targeting a potential mechanism of resistance to TPT, the upregulated DNA repair by overexpressed CHEK1. This approach potentially widens the therapeutic index by preferentially targeting the ovarian cancer-specific dependence on upregulated or activated CHEK1, and allowing increased cancer cell killing at lower doses of each drug. In the future, it may also be possible to incorporate anti-angiogenic agents as well, due to non-overlapping toxicities.

Disrupting DNA repair mechanisms after DNA damaging chemotherapy is a strategy that we have used clinically [23]. Carboplatin was given to induce DNA breaks, and the PARP inhibitor olaparib was administered in order to block repair of single-strand breaks, which progressed to double-strand breaks during DNA replication, causing cell death. This strategy was designed with the goal of achieving a higher therapeutic index in ovarian cancer cells known to harbor defective DNA repair pathways, particularly in cancers with BRCA mutations. Additionally, the increased potency was attained with reduced doses of each drug. In our current study, we achieved the same phenomenon *in vitro*, by co-treating with CHEK1 inhibitor and TPT. This combination synergistically inhibited cellular proliferation at sub-lethal concentrations for each drug given alone. The cytotoxic synergism was accordingly reflected in apoptotic activities and DNA damage response. Interestingly, we found that OV90 and PEO4 cells were resistant to TPT alone, and co-inhibition with TPT and PF477736 resulted in achieving equivalent cellular toxicity with reduced doses of both drugs. However, this co-inhibition still failed to attain complete cellular toxicity in OV90 and PEO4. Further studies are required to know why these two cell lines were so resistant to TPT and even to the co-treatment.

BRCA-mutated cancers tend to have the highest response rates to DNA damaging agents in general. Indeed, our results confirm that the BRCA-mutated cell line PEO1 was the most sensitive of HGS cancers to treatment with TPT or CHEK1 inhibitor alone. Most HGS ovarian cancers, however, do not have BRCA mutations, but do have elevated expression of CHEK1. Thus, combination therapy of CHEK1 inhibitor with DNA damaging agents such as TPT could introduce an analogous strategy for targeting non-BRCA mutated or BRCA-like HGS ovarian cancers.

Another potential marker for potency to CHEK1 inhibitor may be its autophosphorylation. Decreased levels of CHEK1 S296 autophosphorylation have been correlated with response to CHEK1 inhibition in colon, breast, and ovarian cancer cell lines [8,24]. As shown in this study, this marker is expressed in HGS ovarian cancer cell lines at steady states and *CHEK1* gene is over-expressed in nearly all cases of TCGA HGS ovarian cancers [6]. Therefore, this marker should be evaluated at the time of initial diagnosis and in the course of treatment, specifically in recurrent HGS ovarian cancers.

We are currently conducting a phase 2 clinical trial testing single agent CHEK1 inhibitor LY2606368 in women whose cancers are likely to have defects in DNA repair (NCT02203513). The trial will study 3 cohorts of cancers: BRCA-mutated, HGS ovarian cancer, and TNBC. We hypothesize that these types of cancers will be susceptible to CHEK1 inhibition due to their poor ability to repair double strand DNA breaks. The adverse events in this trial have not yet been analyzed. Among many CHEK1 inhibitors under clinical evaluation, an ideal CHEK1 inhibitor to use with TPT for combination treatment would exhibit minimal overlap in toxicity such as myelosuppression. Based on our data, however, it is possible that the anti-cancer effect of the combination will be achieved at lower doses of both agents, which would allow for safe administration of these two drugs, specifically targeted to patients with HGS ovarian cancer.

## Conclusions

Our study shows that TPT synergistically induces cytotoxicity in combination with CHEK1 inhibitor especially in HGS ovarian cancer cells.

## Additional file

Additional file 1:
**Mutation status of** ***TOP1*** **gene in the Cancer Cell Line Encyclopedia.**

## References

[1] McCluggage WG (2011). Morphological subtypes of ovarian carcinoma: a review with emphasis on new developments and pathogenesis. Pathol.

[2] Cancer Genome Atlas Research Network (2011). Integrated genomic analyses of ovarian carcinoma. Nature.

[3] Dasari S, Bernard Tchounwou P (2014). Cisplatin in cancer therapy: molecular mechanisms of action. Eur J Pharmacol.

[4] Slichenmyer WJ, Rowinsky EK, Donehower RC, Kaufmann SH (1993). The current status of camptothecin analogues as antitumor agents. J Natl Cancer Inst.

[5] Lorusso D, Pietragalla A, Mainenti S, Masciullo V, Di Vagno G, Scambia G (2010). Review role of topotecan in gynaecological cancers: current indications and perspectives. Crit Rev Oncol Hematol.

[6] Kim MK, Min DJ, Wright G, Goldlust I, Annunziata CM (2014). Loss of compensatory pro-survival and anti-apoptotic modulator, IKKepsilon, sensitizes ovarian cancer cells to CHEK1 loss through an increased level of p21. Oncotarget.

[7] Goto H, Izawa I, Li P, Inagaki M (2012). Novel regulation of checkpoint kinase 1: Is checkpoint kinase 1 a good candidate for anti-cancer therapy?. Cancer Sci.

[8] Rawlinson R, Massey AJ (2014). GammaH2AX and Chk1 phosphorylation as predictive pharmacodynamic biomarkers of Chk1 inhibitor-chemotherapy combination treatments. BMC Cancer.

[9] Parsels LA, Qian Y, Tanska DM, Gross M, Zhao L, Hassan MC (2011). Assessment of chk1 phosphorylation as a pharmacodynamic biomarker of chk1 inhibition. Clin Cancer Res.

[10] Dent P, Tang Y, Yacoub A, Dai Y, Fisher PB, Grant S (2011). CHK1 inhibitors in combination chemotherapy: thinking beyond the cell cycle. Mol Interv.

[11] Tao ZF, Lin NH (2006). Chk1 inhibitors for novel cancer treatment. Anticancer Agents Med Chem.

[12] Domcke S, Sinha R, Levine DA, Sander C, Schultz N (2013). Evaluating cell lines as tumour models by comparison of genomic profiles. Nat Commun.

[13] Langdon SP, Lawrie SS, Hay FG, Hawkes MM, McDonald A, Hayward IP (1988). Characterization and properties of nine human ovarian adenocarcinoma cell lines. Cancer Res.

[14] Barretina J, Caponigro G, Stransky N, Venkatesan K, Margolin AA, Kim S (2012). The Cancer Cell Line Encyclopedia enables predictive modelling of anticancer drug sensitivity. Nature.

[15] Blasina A, Hallin J, Chen E, Arango ME, Kraynov E, Register J (2008). Breaching the DNA damage checkpoint via PF-00477736, a novel small-molecule inhibitor of checkpoint kinase 1. Mol Cancer Ther.

[16] Aris SM, Pommier Y (2012). Potentiation of the novel topoisomerase I inhibitor indenoisoquinoline LMP-400 by the cell checkpoint and Chk1-Chk2 inhibitor AZD7762. Cancer Res.

[17] Huntoon CJ, Flatten KS, Wahner Hendrickson AE, Huehls AM, Sutor SL, Kaufmann SH (2013). ATR inhibition broadly sensitizes ovarian cancer cells to chemotherapy independent of BRCA status. Cancer Res.

[18] Xiao Y, Ramiscal J, Kowanetz K, Del Nagro C, Malek S, Evangelista M (2013). Identification of preferred chemotherapeutics for combining with a CHK1 inhibitor. Mol Cancer Ther.

[19] Walton MI, Eve PD, Hayes A, Valenti MR, De Haven Brandon AK, Box G (2012). CCT244747 is a novel potent and selective CHK1 inhibitor with oral efficacy alone and in combination with genotoxic anticancer drugs. Clin Cancer Res.

[20] Daud AI, Ashworth MT, Strosberg J, Goldman JW, Mendelson D, Springett G (2015). Phase I dose-escalation trial of checkpoint kinase 1 inhibitor MK-8776 as Monotherapy and in combination with gemcitabine in patients with advanced solid tumors. J Clin Oncol.

[21] Pujade-Lauraine E, Hilpert F, Weber B, Reuss A, Poveda A, Kristensen G (2014). Bevacizumab combined with chemotherapy for platinum-resistant recurrent ovarian cancer: the AURELIA open-label randomized phase III trial. J Clin Oncol.

[22] Vergote I, Schilder RJ, Pippitt CH, Wong S, Gordon AN, Scudder S (2014). A phase 1b study of trebananib in combination with pegylated liposomal doxorubicin or topotecan in women with recurrent platinum-resistant or partially platinum-sensitive ovarian cancer. Gynecol Oncol.

[23] Lee JM, Hays JL, Annunziata CM, Noonan AM, Minasian L, Zujewski JA (2014). Phase I/Ib study of olaparib and carboplatin in BRCA1 or BRCA2 mutation-associated breast or ovarian cancer with biomarker analyses. J Natl Cancer Inst.

[24] Bryant C, Rawlinson R, Massey AJ (2014). Chk1 inhibition as a novel therapeutic strategy for treating triple-negative breast and ovarian cancers. BMC Cancer.

